# Plasma Membrane Repair Is Regulated Extracellularly by Proteases Released from Lysosomes

**DOI:** 10.1371/journal.pone.0152583

**Published:** 2016-03-30

**Authors:** Thiago Castro-Gomes, Matthias Corrotte, Christina Tam, Norma W. Andrews

**Affiliations:** Department of Cell Biology and Molecular Genetics, University of Maryland, College Park, Maryland, 20742, United States of America; UPR 3212 CNRS -Université de Strasbourg, FRANCE

## Abstract

Eukaryotic cells rapidly repair wounds on their plasma membrane. Resealing is Ca^2+-^dependent, and involves exocytosis of lysosomes followed by massive endocytosis. Extracellular activity of the lysosomal enzyme acid sphingomyelinase was previously shown to promote endocytosis and wound removal. However, whether lysosomal proteases released during cell injury participate in resealing is unknown. Here we show that lysosomal proteases regulate plasma membrane repair. Extracellular proteolysis is detected shortly after cell wounding, and inhibition of this process blocks repair. Conversely, surface protein degradation facilitates plasma membrane resealing. The abundant lysosomal cysteine proteases cathepsin B and L, known to proteolytically remodel the extracellular matrix, are rapidly released upon cell injury and are required for efficient plasma membrane repair. In contrast, inhibition of aspartyl proteases or RNAi-mediated silencing of the lysosomal aspartyl protease cathepsin D enhances resealing, an effect associated with the accumulation of active acid sphingomyelinase on the cell surface. Thus, secreted lysosomal cysteine proteases may promote repair by facilitating membrane access of lysosomal acid sphingomyelinase, which promotes wound removal and is subsequently downregulated extracellularly by a process involving cathepsin D.

## Introduction

Ca^2+^ influx through plasma membrane (PM) wounds triggers a rapid repair process that reseals cells within <30 seconds. This mechanism is critical for the survival of eukaryotic cells, which are frequently wounded by mechanical stress [1] or during encounters with pathogens [2][3][4]. Defects in PM repair are associated with muscle pathology, including certain forms of myositis [5] and muscular dystrophy [6–8]. Extensive evidence indicates that Ca^2+^-triggered exocytosis of a peripheral population of lysosomes is an early and essential component of the PM repair process [8–12]. Surprisingly, additional studies revealed that Ca^2+^-dependent lysosomal exocytosis is followed by massive membrane internalization [13, 14], which removes damaged regions of the PM and promotes resealing [15–17]. Membrane budding and extracellular shedding were also proposed as a cell resealing mechanism [18], and recently the ESCRT complex was implicated in the removal of small wounds from the PM [19]. These findings introduced an important new concept: PM repair involves the direct removal of damaged portions of the membrane, and not simply patching of the wound with intracellular membranes [20]. Thus, it is now important to understand how the wounded PM is remodeled during the lesion removal process, and what are the molecular players in this process.

To date, most studies of PM repair focused on intracellular events, triggered by the massive Ca^2+^ influx that occurs in wounded cells. Ubiquitously expressed Ca^2+^-dependent cytosolic proteins such as annexins, calpains and transglutaminases have been implicated in mechanisms that promote cellular survival, and in some cases were shown to form large complexes in association with the cytoplasmic side of PM wounds—a process that may reduce cytosol loss and/or remodel the inner leaflet of the PM to facilitate resealing [21–26]. In muscle fibers and in a few additional tissues, specialized intracellular proteins such as dysferlin and MG53 also participate in PM repair [6, 7]. The cytosolic region of dysferlin contains several Ca^2+^-binding C2 domains, and recent evidence suggests that it functions as a PM Ca^2+^ sensor that promotes lysosomal exocytosis [27]. This notion of a PM Ca^2+^-sensing molecule complements previous results showing that Syt VII, a ubiquitously expressed member of the synaptotagmin family of Ca^2+^ sensors, is present on the membrane of lysosomes where it regulates exocytosis [28–30] and PM repair [5].

Cytosolic Ca^2+^-dependent proteins and lysosomal exocytosis have been largely thought to facilitate PM resealing by generating a membrane patch or reducing membrane tension, through the addition of intracellular membrane to the cytoplasmic side of the injured PM [31, 32]. However, a role for the hydrolases present in the lumen of lysosomes has recently emerged, with the demonstration that purified acid sphingomyelinase (ASM) promotes endocytosis and wound removal when added extracellularly, rescuing the PM repair defect of ASM-deficient cells [33]. These findings revealed for the first time that lysosomal exocytosis releases factors that can remodel the external surface of wounded cells, promoting repair. This new insight led us to investigate whether lysosomal proteases released during cell injury also participate in PM resealing.

Lysosomes contain about 50 acid hydrolases involved in bulk degradation of substrates, pro-protein processing, antigen processing, degradation of the extracellular matrix and initiation of apoptosis [34]. The acidic optimum pH of most lysosomal enzymes has been largely interpreted as evidence that their primary site of action is inside the lysosomal compartment. However, evidence has been accumulating in several systems indicating that transient acidified conditions can be generated extracellularly, particularly at sites of lysosomal exocytosis close to the PM [35, 36]. In this study we investigated the role of major classes of lysosomal proteases in cell resealing, and found that some of these enzymes are active extracellularly shortly after wounding and participate in the regulation of PM repair.

## Materials and Methods

### Cell Culture

HeLa (ATCC) and NRK (ATCC) cells were cultivated at 37°C/5% C0_2_ in high glucose DMEM (Dulbecco’s Modified Eagle’s Medium) (Lonza) containing 10% FBS (fetal bovine serum) and antibiotics (penicillin/streptomycin) (Gemini). Cells were plated 48 h before the experiments to obtain sub-confluent cell monolayers in 35 mm glass-bottom microwell dishes (MatTek), 35 or 100 mm plastic dishes (Cellstar), or glass coverslips.

### Propidium Iodide (PI) Exclusion Assays

Histidine-tagged Streptolysin-O (SLO) carrying a cysteine deletion that eliminates the need for thiol activation (provided by R. Tweeten University of Oklahoma, Norman, OK) was expressed and purified as described in [13] and stored in 1 mg/ml aliquots at -80°C. For wounding with SLO, NRK or HeLa cells were trypsinized (0.25% trypsin at 37°C for 5 min) and split in two different suspensions in DMEM (with or without Ca^2+^) at the concentration of 6 x 10^5^ cells/ml. 500 μl of each cell suspension were incubated on ice with the indicated concentration of SLO for 5 min. To induce pore-formation the cells were transferred to a 37°C water bath for the indicated time. For mechanical wounding (scrape) NRK or HeLa cells were cultured in 30 mm dishes at 80% confluence for 48 h, washed 3 x with cold Ca^2+^-free DMEM, 1 ml of 37°C pre-warmed DMEM (with or without Ca^2+^) was added, and cells were quickly scraped in the presence or not of the indicated inhibitors. After scraping, the cells were gently re-suspended with a large tip pipette and further incubated at 37°C for 2 min. To assess PM repair cells were transferred to a 4°C water bath and 50 μg/ml PI (Sigma-Aldrich) was added to stain the nuclei of permeabilized cells. After 5 min the cell population was analyzed by flow cytometry (FACS Canto II, BD Biosciences). The percentage of repair was calculated from the fraction of PI negative cells within the gated region (dashed lines) minus the fraction of PI negative cells detected in the same gated region in the absence of Ca^2+^ (non-wounded cells).

### Time-Lapse Live Imaging of FM1-43 Influx

Sub-confluent HeLa or NRK cells plated on glass-bottom dishes (MatTek) were pre-incubated with SLO for 5 min at 4°C and transferred to a LiveCell System chamber (Pathology Devices) at 37°C with 5% CO_2_. After selecting a field to image, pre-warmed DMEM with or without Ca^2+^ and 4 μM FM1-43 (Invitrogen) was added to the dish. Spinning disk confocal images were acquired for 4 min at 1 frame/6 or 10 s using an UltraVIEW VoX system (PerkinElmer) attached to an inverted microscope (Eclipse Ti; Nikon) equipped with a camera (C9100-50; Hamamatsu Photonics) and a 40x NA 1.3 objective (Nikon). Quantitative analysis of intracellular fluorescence was performed using Volocity Suite (PerkinElmer).

### Protease Inhibitors

To determine the impact of proteases in PM repair, PI exclusion and FM1-43 influx assays were performed in the presence of the following protease inhibitors: the human broad protease inhibitor α-2-macroglobulin (R&D Systems), the inhibitor of cysteine proteases E64 (Sigma-Aldrich), the inhibitor of aspartyl proteases pepstatin-A (Sigma-Aldrich) and the inhibitor of serine proteases AEBSF (Sigma-Aldrich). The drugs were present only during the short incubation period between wounding and PM repair. A cocktail of protease inhibitors (Pierce Protease Inhibitor Mini Tablets, Thermo Scientific) containing AEBSF, aprotinin, bestatin, E64, EDTA, leupeptin and pepstatin-A was used in some experiments, following the manufacturer’s instructions.

### Proteolytic De-Quenching of DQ-BSA

HeLa cells (2 x 10^5^ cells in 500 μl of DMEM) treated or not with SLO were incubated for 2 min at 4°C (controls) or 37°C (SLO wounding and PM repair) in the presence of 50 μg/ml red DQ Red BSA (Invitrogen) in PBS with Ca^2+^. After 1 min on ice 250 μl of each cell suspension were transferred to a flat-bottom 96-well plate and fluorescence was determined using the ELISA reader following manufacture’s instruction. NRK cells were cultured to sub-confluency on MatTeck glass bottom dishes, the medium was replaced with PBS with or without Ca^2+^, and dishes were placed in an environmental chamber at 37°C on a Deltavision Elite Deconvolution microscope (GE Healthcare). 1 ml of PBS with or without Ca^2+^ containing DQ-BSA (0.1 mg/ml final concentration) and SLO (50 ng/ml final concentration) was added to the cells before imaging. Cells were imaged as a Z-stack of 30 sections every 12 s for several min and the movie was processed using the deconvolution function of SoftWoRx (GE Healthcare). Images were processed for contrast and level and exported as TIFF file, and the mean fluorescence intensity levels were determined for each field using Volocity suite (PerkinElmer). Images are shown as extended focus of all 30 optical sections.

### Biotinylation and Detection of Cell Surface Proteolysis

Sub-confluent NRK cells cultured for 48 h in 10 cm dishes were washed 3 x in PBS and incubated with 10 ml of 0.2 mg/ml EZ-Link Sulfo-NHS-LC-Biotin (Thermo Scientific) in PBS pH 8) at 4°C for 30 min. After biotinylation the cells were washed 3 times with cold PBS 100 mM glycin and 3x with PBS. The cells were then incubated with SLO on ice in Ca^2+^-free PBS for 5 min followed by pre-warmed DMEM with or without Ca^2+^ for 30 s in a 37°C water bath in the presence or not of the indicated protease inhibitors. The supernanant media was removed, placed on ice with complete protease cocktail inhibitor, centrifuged for 10 min at 200 x *g* to remove any detached cells and then centrifuged again at 100,000 x *g* for 1 h at 4°C. The supernatant was concentrated 20 x in a 3 kDa Amicon Ultra filter unit and processed for SDS-PAGE using reducing sample buffer and boiling for 5 min. After SDS-PAGE and transfer the membrane was blocked with 5% dried milk for 1 h and incubated overnight with 1:200 streptavidin-HRP (Pierce) in 5% dried milk solution. Detection was performed using Clarity western blot ECL substrate (Bio-Rad) and images were acquired using a Fuji LAS-3000 system with Image Reader LAS-3000 software (Fuji).

### Proteinase-K Treatment

NRK cells cultured for 48 h were trypsinized, washed and incubated at RT with 2.5–150 μg/ml proteinase-K (Sigma-Aldrich) in DMEM with Ca^2+^ for 5 min. 3 x 10^5^ cells were used per condition. To remove the protease cells were washed 3 x in cold Ca^2+^-free PBS and then incubated with SLO followed by PI exclusion/FACS PM repair assays. To assess the effect of proteinase K on Ca^2+^-free repair induced by sphingomyelinase, cells were cultured, trypsinized, treated with 50 μg/ml proteinase K and washed in cold Ca^2+^-free DMEM. 3 x 10^5^ cells were then resuspended in 500 μl of Ca^2+^-free DMEM and incubated with 15 ng/ml SLO (a low concentration of SLO was necessary to detect repair induced by SM in the absence of Ca^2+^) for 5 min at 4°C before addition of 10 μU/ml purified *Bacillus cereus* sphingomyelinase (Sigma). Cells were then warmed to 37°C for 5 min, followed by PI exclusion/FACS PM repair assays as previously described.

### Detection of Secreted Enzymes

NRK or HeLa subconfluent monolayers were washed 3 x with Ca^2+^-free PBS, incubated with SLO on ice for 5 min, washed and kept on ice in the same buffer. To induce pore-formation and trigger exocytosis the medium was aspirated and 250 μl (in 35 mm culture dishes for cathepsin exocytosis) or 2 ml (100 mm culture dishes for ASM exocytosis) of pre-warmed PBS containing Ca^2+^ was gently added to the cells followed by incubation in a 37°C water bath for the indicated time. The supernatant was removed and kept on ice after addition of a protease inhibitor cocktail (Thermo Scientific) for acid sphingomyelinase detection, or without inhibitors for detection of lysosomal proteases. The collected supernatant was centrifuged at 200 x *g* for 5 min to remove any detached cells and analyzed with fluorimetric kits for the detection of cathepsin B (Abcam Cat. #ab65300), cathepsin L (Abcam Cat. #ab65306), cathepsin D (Abcam Cat. #ab65302) and acid sphingomyelinase (Invitrogen Cat. #A12220), following the manufacturer’s instructions. Cathepsins B and L activities were determined using the preferred cathepsin-B (Ac-RR-AFC) or L (AC-FR-AFC) substrates labeled with amino-4-trifluoromethyl coumarin (AFC), as provided by the manufacturer. When cleaved by each enzyme, the synthetic substrate releases the free AFC that can be quantified using a fluorescence plate reader (Ex/Em = 400/505 nm). Cathepsin D activity was measured using the preferred cathepsin-D substrate GKPILFFRLK(Dnp)-D-R-NH_2_ labeled with 7-methoxycoumarin-4-yl-acetyl (MCA), which is also released upon proteolytic cleavage and detected fluorometrically (Ex/Em = 328/460 nm). For cathepsins B, L and D detection, 50 ul of culture supernatant containing the exocytosed lysosomal enzymes were incubated in a 96 well clear bottom ELISA plate with 50 ul of the reaction buffer provided by the manufacturer containing each substrate (final concentration 200 μM for the cathepsin B and L assays, and 20 μM for the cathepsin D assay). Substrate digestion was continuously monitored at 37°C and quantifications were performed within the linear phase of each reaction. For the detection of ASM a two-step enzyme-coupled assay was used to indirectly detect the activity of the lipase using 10-acetyl-3,7-dihydroxyphenoxazine (Amplex® Red reagent), a sensitive fluorogenic probe for H_2_O_2_. ASM was distinguished from neutral sphingomyelinase by performing the assay in two steps. In the first step, hydrolysis of the sphingomyelin substrate by ASM present in the samples was performed in 50 mM sodium acetate buffer pH 5.0 (at a 1:1 ratio with the exocytosed material obtained in PBS or cell lysate). These acidic conditions are optimal for detection of ASM and not neutral sphingomyelinase activity. After sphingomyelin digestion for 1 h, an equal volume of 100 mM Tris-HCL pH 8.0 was added to elevate the pH and allow action of the enzymes involved in the second step of the assay (a pool of alkaline phosphatase, choline oxidase and horseradish peroxidase provided by the manufacturer). Following the action of alkaline phosphatase, which hydrolyses phosphorylcholine, choline is oxidized by choline oxidase to betaine and H_2_O_2_. In the presence of horseradish peroxidase, H_2_O_2_ reacts with the Amplex® Red reagent generating fluorescence which was quantified using a fluorescence plate reader (Ex/Em = 571/585 nm). To determine the total enzymatic activity, 1 x 10^6^ cells detached by tripsinization were lysed in 50 μl of the lysis buffer provided by the fluorometric kit manufacturer, the protein content was determined (BCA protein assay, Thermo Scientific) and 25 μg was used to assay enzymatic activity as described above. To evaluate the effect of proteases on exocytosed ASM, E64, pepstatin-A or AEBSF were added to the medium during the 37°C incubation period required for PM repair, at the concentrations indicated in each experiment. To verify the specificity of the enzymatic kits used, HeLa cells were transfected with the siRNA for each enzyme analysed (cathepsin B, L, D and ASM), and enzymatic activity was evaluated after 24 and 48 h. Sphingomyelinase assays were performed at both pH 5.0 and pH 7.4, to ensure that only ASM was specifically detected.

### Detection of PM Associated Extracellular ASM

NRK or HeLa cell monolayers in 100 mm dishes were treated with SLO and inhibitors as described above. After harvesting the supernatant media the cell monolayer was placed on ice and immediately washed for 5 s with 2 ml of cold acid wash solution (Ca^2+^-free PBS, 25 mM acetic acid pH 4.2) to remove membrane-bound ASM. The samples were placed on ice with a protease inhibitor cocktail and the pH was neutralized using Tris-HCl pH 8.0. The samples were centrifuged for 10 min at 200 x *g* to remove detached cells, filtered through a 0.2 mm filter, concentrated 20 x using 3 kDa Amicon Ultra filter units and analyzed by SDS-PAGE and Western Blot as described below.

### Immunofluorescence and Western Blot

Immunoblot and immunofluorescence assays were performed using the following antibodies: rabbit polyclonal anti-ASM (Abcam ab83354), rabbit polyclonal anti-cathepsin L (Abcam ab58991), rabbit polyclonal anti-cathepsin B (Calbiochem PC-41), goat anti-cathepsin D (Santa Cruz sc-6486) and mouse anti-tubulin (Sigma T6199) for loading controls. Protein samples were prepared with reducing sample buffer, boiled, separated on 10% SDS-PAGE or 4–20% gradient polyacrylamide gels (Bio-Rad) and blotted on nitrocellulose membranes (Bio-Rad) using the Trans-Blot transfer system (Bio-Rad) overnight at 15 V, or for 1 h 30 min at 100 V. The membranes were blocked for 1 h in 5% milk solution. After incubation with the primary antibodies and peroxidase conjugated secondary antibodies, detection was performed using ECL Substrate (Thermo Scientific) or Clarity western blot ECL substrate (Bio-Rad) and a Fuji LAS-3000 Imaging System with Image Reader LAS-3000 software (Fuji). Samples were normalized for protein concentration using a BCA Protein assay kit (Thermo Fisher Scientific). For immunofluorescence, NRK cells were treated with 100 ng/ml SLO at 4°C for 5 min and incubated at 37°C for the indicated time. Cells were fixed in 4% paraformaldehyde for 10 min and washed 3 x in PBS followed by incubation for 45 min at room temperature in PBS containing 2% BSA. Cells were then incubated for 2 h with anti-ASM antibodies diluted 1:500 in PBS 1% BSA, washed in PBS, followed by 1 h incubation with secondary anti-rabbit antibodies conjugated to Alexa Fluor 488 (Invitrogen). Coverslips were then mounted using ProLong® Gold antifade reagent (Invitrogen) and imaged using a Nikon epifluorescence microscope.

### siRNA Silencing

HeLa cells at 40% confluence were transfected in reduced serum DMEM 2.5% FBS without penicillin/streptomycin using Lipofectamine RNAiMax (Invitrogen) and 960 pmol of cathepsin B (GGA UCA CUG UGG AAU CGA AUC AGA A), cathepsin L (GGC UAC GGA UUU GAA AGC ACA GAA U), cathepsin D (CCA UUC CCG AGG UGC UCA AGA ACU A), SMPD1 (GCCCUGCCGUCUGGCUACUCUUUGU) or medium GC content control stealth siRNA duplexes, according to the manufacturer’s instructions (Invitrogen). After 12 h of incubation the medium was removed, replaced by DMEM 10% FBS + penicillin/streptomycin, and cells were further cultivated for 36, 48 or 72 h. The degree of protein expression after silencing was determined using the fluorimetric enzymatic activity assays described above.

## Results

### Extracellular Proteolysis during the First Seconds after Injury Promotes PM Resealing

**Fig 1:**
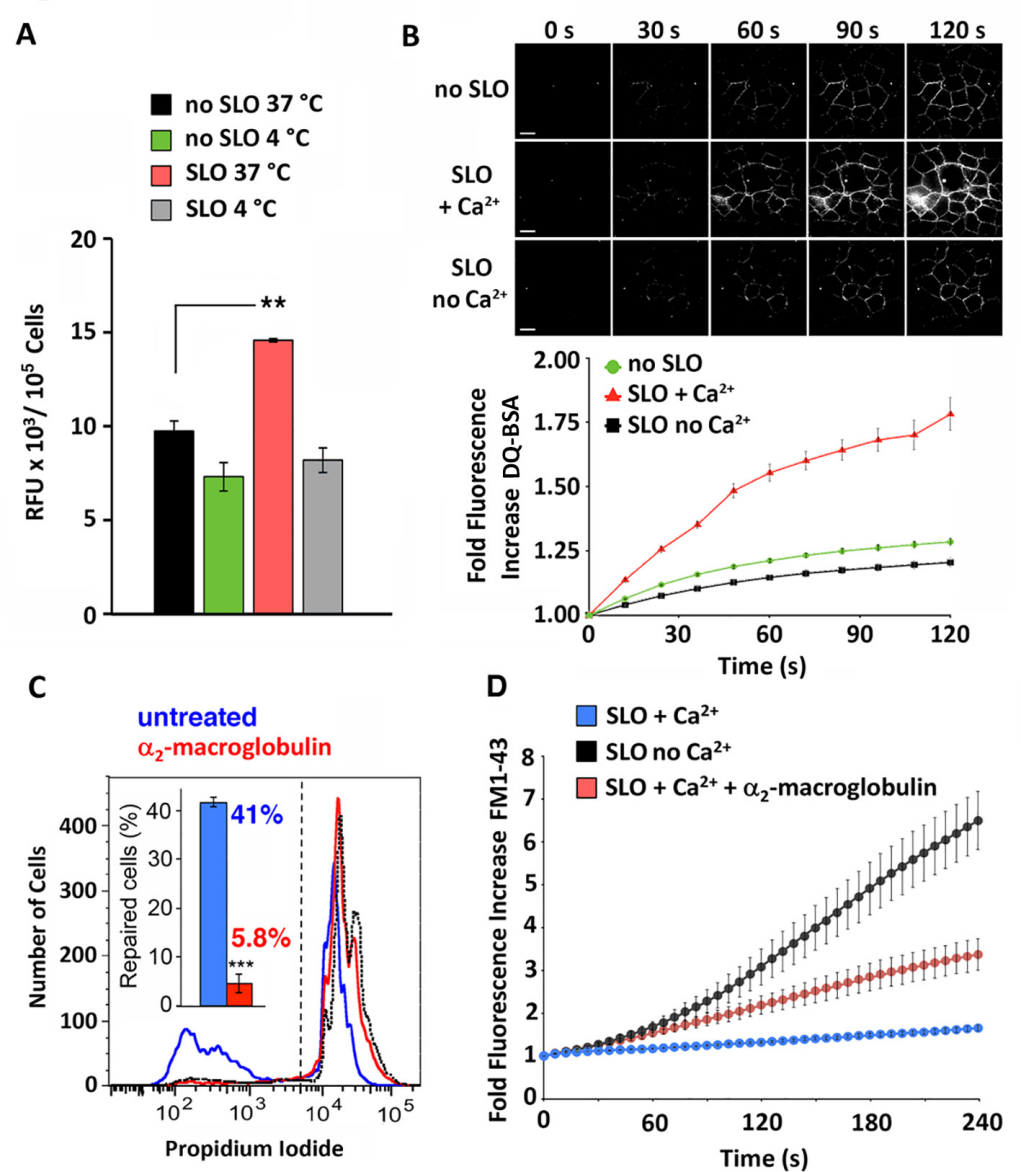
Rapid extracellular proteolysis triggered by SLO wounding is required for PM repair. **(A)** Cleavage of extracellularly added DQ-BSA during PM wounding and repair. HeLa cells treated or not with 200 ng/ml SLO were incubated with DQ-BSA for 2 min at the indicated temperature, followed by measuring the de-quenching generated fluorescence. The data represent the mean +/- SD of triplicate assays. ** P = 0.00297, Student’s t test. **(B)** De-quenched DQ-BSA associated with the surface of wounded cells. NRK cells treated or not with 50 ng/ml SLO in the presence or absence of Ca^2+^ were incubated with DQ-BSA followed by fluorescence deconvolution imaging (top panel). Bars = 10 μm. The mean cell-associated fluorescence intensity was determined and expressed as fold increase relative to the starting level (bottom panel). The data represent the mean +/- SD of fluorescence intensity values associated with 24–26 individual cells, and are representative of two independent experiments. **(C)** FACS quantification of PI staining in NRK cells permeabilized with SLO (200 ng/ml) in the presence (red) or absence (blue) of the protease inhibitor alpha-2-macroglobulin (20 μg/ml). The dotted histogram shows the Ca^2+^-free permeabilization control, which determined the gating (dashed line). The inset shows the percentage of cells that excluded PI after 2 min at 37°C. The data represent the mean +/- SD of three independent experiments. **(D)** Time-lapse live imaging of FM1-43 influx into NRK cells exposed to SLO (350 ng/ml) in the presence or absence of Ca^2+^ and the protease inhibitor alpha-2-macroglobulin (20 μg/ml). The data represent the mean +/- SEM of 27–52 cells per condition.

Our initial studies detected extracellular proteolysis at early stages of the PM repair process, in cells wounded by the pore-forming toxin streptolysin O (SLO). In the presence of Ca^2+^, SLO pore formation at 37°C resulted in extracellular de-quenching of the proteolytically activated fluorogenic substrate DQ-BSA. Proteolysis-dependent fluorescence was detected on HeLa cells in suspension and also on attached NRK cells, taking advantage of the ability of DQ-BSA to bind extracellularly to the cell surface (Fig 1A and 1B). When analyzed by live fluorescence microscopy, a marked increase in DQ-BSA fluorescence was increasingly observed on the surface of NRK cells wounded with SLO in the presence of Ca^2+^, a condition that allows lysosomal exocytosis and PM repair [9] (Fig 1B). In both cell types, proteolytic de-quenching of DQ-BSA was significantly lower under conditions that do not trigger lysosomal exocytosis, such as in the absence of pore formation (no SLO, or SLO 4°C) or in the absence of Ca^2+^. The PM-associated DQ-BSA proteolysis occurred rapidly, being detectable just a few seconds after cell injury (Fig 1B). These results indicate that proteases are released from cells and are active extracellularly during the process of PM wounding and repair.

To confirm the extracellular location of the proteolytic events and to determine if they play a role in PM repair, we added the protease inhibitor α-2-macroglobulin (A2M) to the medium during assays. A2M is a ~780 kDa plasma proteinase inhibitor composed of four covalently linked subunits joined by disulfide bonds, which inhibits endopeptidases of any class and nearly any specificity [37]. The presence of A2M extracellularly during the first 2 minutes after cell wounding had a strong inhibitory effect on PM repair, as observed in assays measuring propidium iodide (PI) (Fig 1C) or FM1-43 (Fig 1D) influx. In both assays, dye entry reflects PM permeability, and repair is assessed by the degree by which this process is blocked [13]. No inhibition in PM repair was observed when A2M was previously inactivated by boiling. Considering the very large size of A2M, it is unlikely that it was able to cross the SLO pores (~30 nm) and enter cells during the few seconds during which PM repair occurs (Fig 1D). Thus, these results suggest that extracellular proteolysis triggered by cell injury facilitates PM repair.

**Fig 2:**
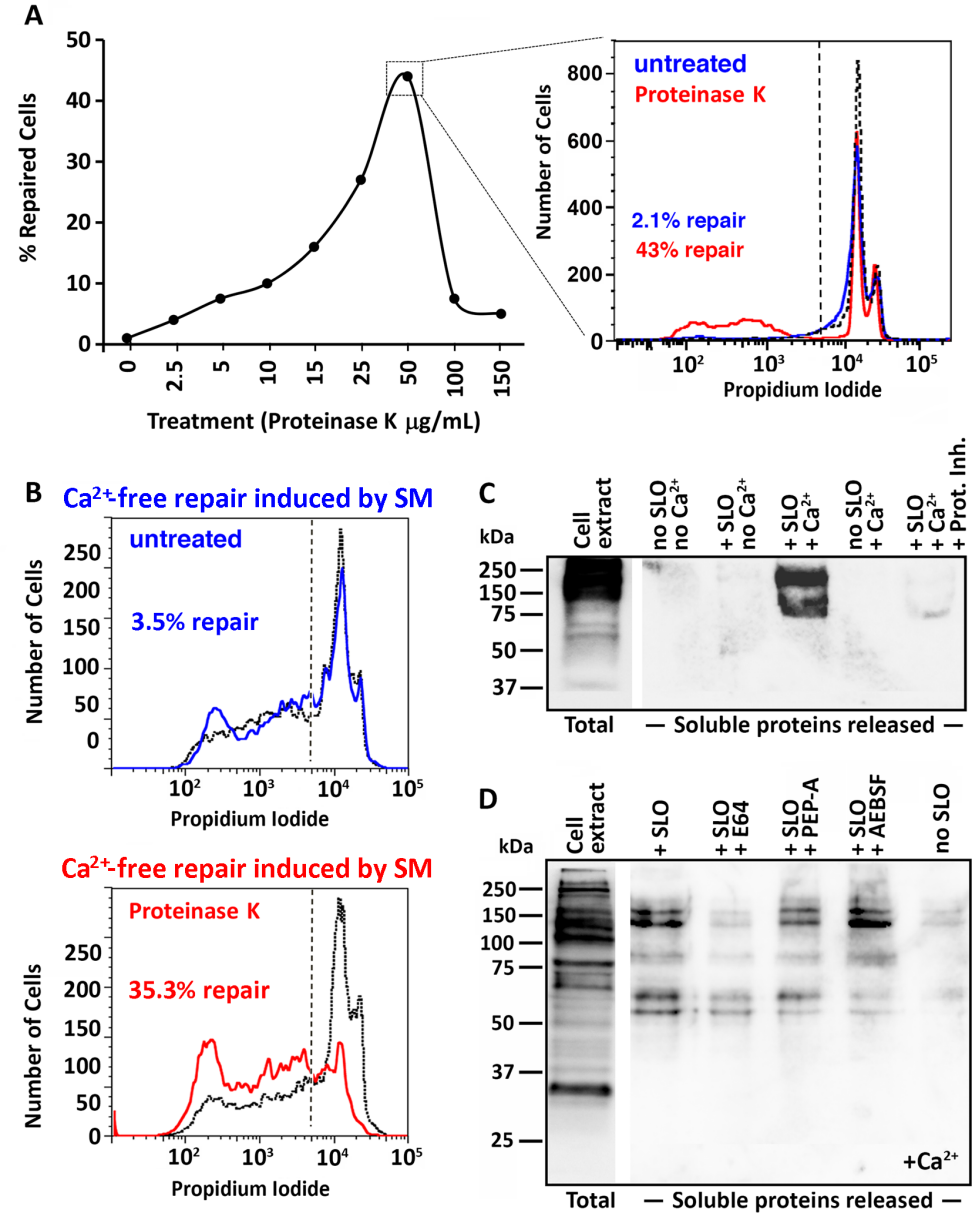
Extracellular proteolysis releases surface proteins and promotes PM repair. **(A)** Effect of proteinase K treatment on Ca^2+-^dependent PM repair. NRK cells pre-treated with increasing concentrations of proteinase K were permeabilized with SLO (150 ng/ml), incubated at 37°C for 5 min, stained with PI and analyzed by FACS. The inset on the right shows one example of PI quantification for cells untreated (blue) or pre-treated with 50 μg/ml proteinase K (red). The dotted histogram shows the Ca^2+^-free permeabilization control, which determined the gating (dashed line). The data are representative of three independent experiments. **(B)** Effect of proteinase K treatment on SM-induced Ca^2+^-free PM repair. NRK cells treated (red) or not (blue) with 50 μg/ml proteinase K were permeabilized with SLO (15 ng/ml), incubated at 37°C for 5 min in Ca^2+^-free DMEM containing 10 μU/ml SM, stained with PI and analyzed by FACS. The dotted black histogram shows the Ca^2+^-free permeabilization control in the absence of sphingomyelinase; the data are representative of five independent experiments. (C, D) Biotinylated surface proteins released in soluble form during SLO wounding and repair. NRK cells were biotinylated at 4°C and permeabilized or not with SLO (100 ng/ml) in the presence or not of Ca^2+^ and containing or not a cocktail of protease inhibitors (Prot. Inh.), 100 μM E64, pepstatin-A (PEP-A) or AEBSF for 30 s, followed by collection of the supernatant, centrifugation at 100,000 g and analysis by Western blot with streptavidin-HRP. A diluted sample of the total cell extract before SLO wounding is shown on the left. The data are representative of two (C) or three (D) independent experiments.

To further evaluate whether extracellular proteolysis improved the ability of cells to undergo PM repair, we pre-exposed NRK cells to exogenous proteinase K briefly (5 min) and subsequently determined the ability of these cells to repair SLO wounds. In these experiments we used high concentrations of SLO that normally result in low levels of resealing (as indicated in Fig 2A, ~2% repair), in order to determine if there was an improvement after proteinase K treatment. FACS/PI exclusion assays revealed that 2.5–50 μg/ml proteinase K rescued cells from a non-repair condition to up to 43% repair, in a dose-dependent manner (Fig 2A). Above 100 μg/ml proteinase K the fraction of cells that were able to reseal dropped abruptly, indicating a possible deleterious effect of the protease on the repair machinery at high concentrations. As assessed by PI staining assays in the absence of Ca^2+^, proteinase K treatment did not affect the level of SLO permeabilization, in agreement with the role of cholesterol as a receptor for this toxin [38]. To test the hypothesis that extracellular proteolysis may facilitate the access of ASM to the cell surface after exocytosis from lysosomes, we took advantage of the earlier observation that exogenously added purified *B*. *cereus* sphingomyelinase (SM) can promote PM repair in the absence of extracellular Ca^2+^, a condition not permissive for lysosomal exocytosis [39]. As expected, NRK cells permeabilized with SLO in Ca^2+^-free medium showed a small increase in the number of PI-negative cells after treatment with SM (Fig 2B, upper panel). When the cells were pre-treated with 50 μg/ml proteinase K and then permeabilized with SLO and exposed to SM under the same Ca^2+^-free conditions, the fraction of PI-negative cells (% repair) was enhanced about ten fold (Fig 2B, lower panel). Thus, cleavage of extracellular proteins by proteinase K appears to facilitate the action of sphingomyelinase on the PM, to promote resealing.

To verify whether extracellular proteolysis triggered by wounding targeted PM proteins under physiological conditions (in cells not pre-treated with proteinase K), we biotinylated proteins exposed on the surface of NRK cells prior to wounding and repair assays. Initial analysis of the supernatant media revealed that a substantial amount of biotinylated proteins were released in soluble form, but only when cells were permeabilized with SLO in the presence of Ca^2+^ (Fig 2C–compare + SLO no Ca^2+^ with + SLO + Ca^2+^). This result indicates that the removal of surface proteins is not triggered by wounding *per se*, but is a consequence of the resealing process that only occurs in the presence of Ca^2+^ (a condition that triggers rapid exocytosis of lysosomes). Further analysis using gradient gels for better SDS-PAGE resolution revealed biotinylated protein fragments of different sizes in the supernatant of cells treated with SLO + Ca^2+^. This release was markedly reduced when the assay was performed in the presence of the cysteine protease inhibitor E64. Smaller and more varied degrees of inhibition (differentially affecting proteins of high and low molecular mass) were observed with the aspartyl protease inhibitor pepstatin-A (PEP-A) and the serine protease inhibitor AEBSF (Fig 2D). Thus, proteins exposed on the cell surface are proteolytically removed shortly after PM injury under conditions permissive for lysosomal exocytosis and PM repair, and extracellularly active cysteine proteases appear to play a role in this process.

### Cysteine Protease Inhibition Reduces, and Aspartyl Protease Inhibition Enhances PM Repair

**Fig 3:**
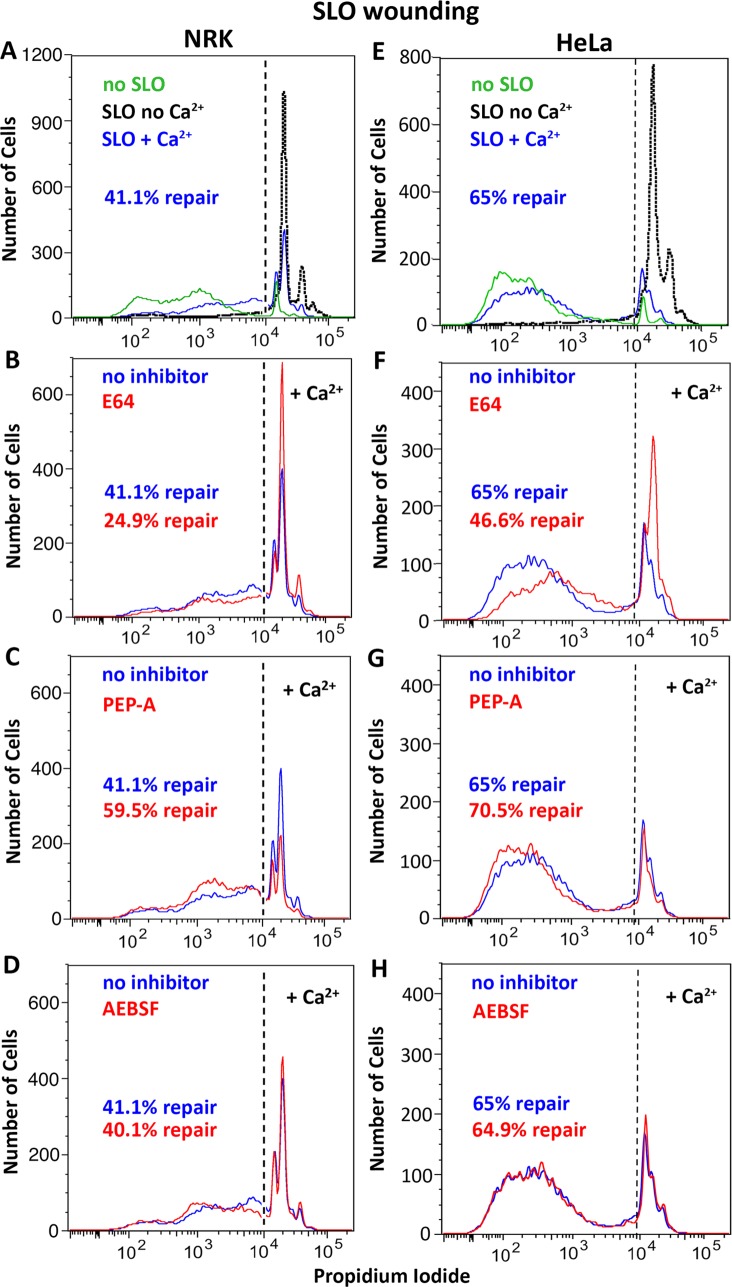
Protease inhibitors rapidly modulate the repair of SLO wounds. NRK (left column) or HeLa (right column) cells were wounded with SLO (NRK 150 ng/ml, HeLa 250 ng/ml) and PM repair assays were performed in the absence **(A, E)** or in the presence of the following protease inhibitors: **(B, F)** E64 (100 μM); **(C, G)** Pepstatin-A (PEP-A,100 μM); **(D, H)** AEBSF (100 μM). After a resealing period of 2 min at 37°C the cell population was stained with PI and analyzed by FACS. In (A, E) the green histograms show the FACS profile of not wounded cells, and the dotted black histograms show the Ca^2+^-free permeabilization controls, which determined the gating (dashed line). The data are representative of at least three independent experiments.

**Fig 4:**
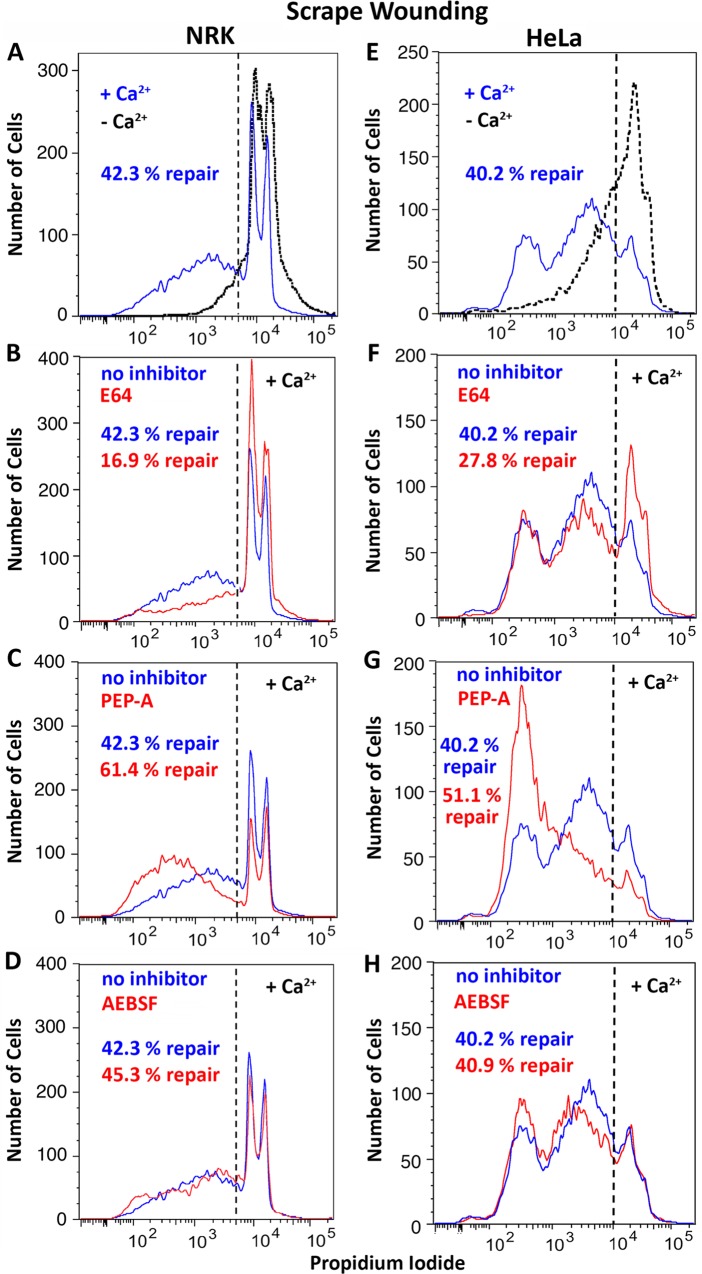
Protease inhibitors rapidly modulate the repair of mechanical wounds. NRK (left columns) or HeLa (right columns) cells were wounded by scraping and PM repair assays were performed in the absence **(A, E)** or in the presence of the following protease inhibitors: **(B, F)** E64 (100 μM); **(C, G)** Pepstatin-A (PEP-A,100 μM); **(D, H)** AEBSF (100 μM). After a resealing period of 2 min at 37°C the cell population was stained with PI and analyzed by FACS. The dotted black lines in (A, E) show the Ca^2+^-free permeabilization controls, which determined the gating (dashed line). The data are representative of at least three independent experiments.

Next, we investigated the effect of cysteine, aspartyl and serine protease inhibitors on the ability of cells to reseal their PM. In the presence of the cysteine protease inhibitor E64, the ability of cells to repair wounds triggered by SLO was reduced compared to controls in both NRK (Fig 3A and 3B) and HeLa cells (Fig 3E and 3F). Interestingly, the aspartyl protease inhibitor pepstatin-A had the opposite effect, facilitating PM repair in both cell types (Fig 3A–3C and 3A–3G). Under the same conditions, the serine protease inhibitor AEBSF had no significant effect (Fig 3A–3D and 3A–3H). Very similar results were obtained when the cells were wounded by scraping from the dish, indicating that the observed effect of protease inhibitors is not restricted to wounding with the pore-forming protein SLO (Fig 4). A similar effect of the inhibitors was also observed using a live microscopy kinetic assay of FM1-43 influx (S1 Fig).

Given that E64 and pepstatin-A cannot efficiently cross membranes and were in contact with cells for only a short period of time (2 min), it is likely that these inhibitors exerted their action on PM repair extracellularly. The short interaction period between the inhibitors and their targets may also explain why concentrations in the μM range were required to observe a measurable effect of E64 and pepstatin-A on PM repair. Our results suggest that one or more aspartyl proteases released from wounded cells may interfere with PM resealing, whereas cysteine proteases facilitate the process. Given that cysteine protease inhibitors inhibit the release of PM proteins triggered by wounding (Fig 2C and 2D), our findings suggest that cysteine proteases released from lysosomes promote PM repair through their extracellular action on cell surface-associated proteins. This finding is in agreement with earlier studies showing that cysteine proteases, a major class of lysosomal hydrolases, are involved in remodeling and degradation of the extracellular matrix [40, 41].

### Active Cathepsins B, L and D Are Released Extracellularly during PM Injury and Have Opposing Effects on PM Repair

**Fig 5:**
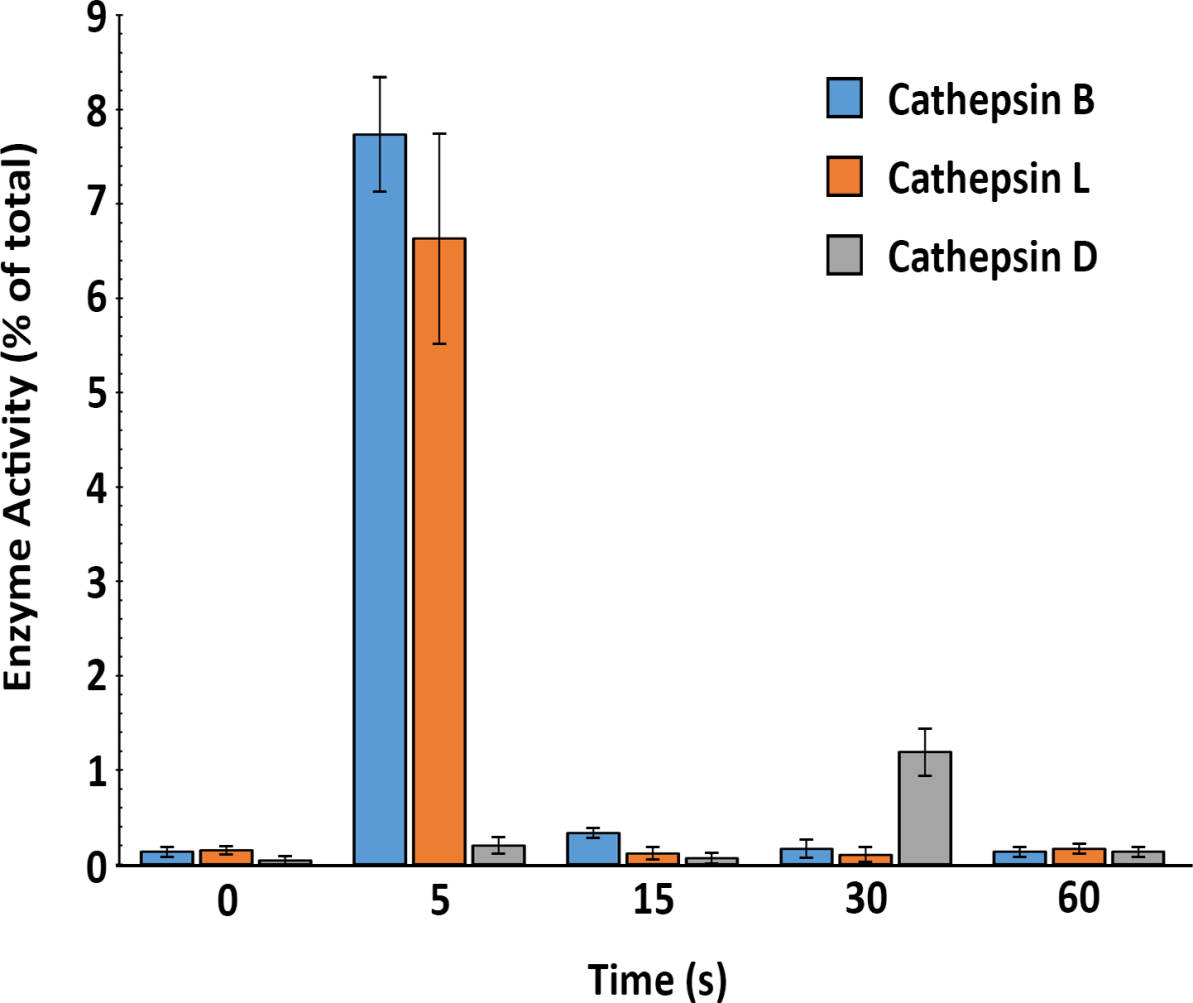
Active lysosomal proteases are released with differential kinetics during PM wounding and repair. NRK cells were pre-incubated on ice with SLO (100 ng/ml) followed by addition of Ca^2+^-containing media pre-warmed at 37°C to trigger pore-formation and lysosomal exocytosis. The supernatant was collected at the indicated periods of time and analyzed for activity of the cysteine proteases cathepsins B and L and the aspartyl protease cathepsin D. Results are expressed as percentage of the total activity present in the cells (determined by assaying enzymatic activity in whole cell lysates), and correspond to the mean +/- SD of three independent experiments.

Ca^2+^ entry into wounded cells triggers rapid exocytosis of lysosomes, an event required for PM repair [9]. Our detection of extracellular proteolytic activity a few seconds after cell wounding (Fig 1B) suggested that lysosomes might be the source of the proteases regulating PM repair. To better understand this process, we followed the activity of the abundant lysosomal hydrolases cathepsin B and L (major lysosomal cysteine proteases) and cathepsin D (major lysosomal aspartyl protease) in the cell supernatant, in the first few seconds after cell wounding. NRK cells were pre-incubated with SLO on ice, and pore-formation was synchronously triggered by the addition of pre-warmed Ca^2+^-containing media. Enzymatic activity was then assessed in the extracellular medium at different time points using specific fluorogenic substrates. The specificity of the substrates for each protease was confirmed using siRNA-mediated knockdown assays (S2 Fig). Both cell types secreted large amounts of cathepsin B and L (5–7% of the total cellular content) just 5 seconds after pore-formation, at the beginning of PM repair process. Smaller amounts of cathepsin D activity were detected (~1% of the total cellular content) after a delay, about 30 seconds after wounding (Fig 5).

**Fig 6:**
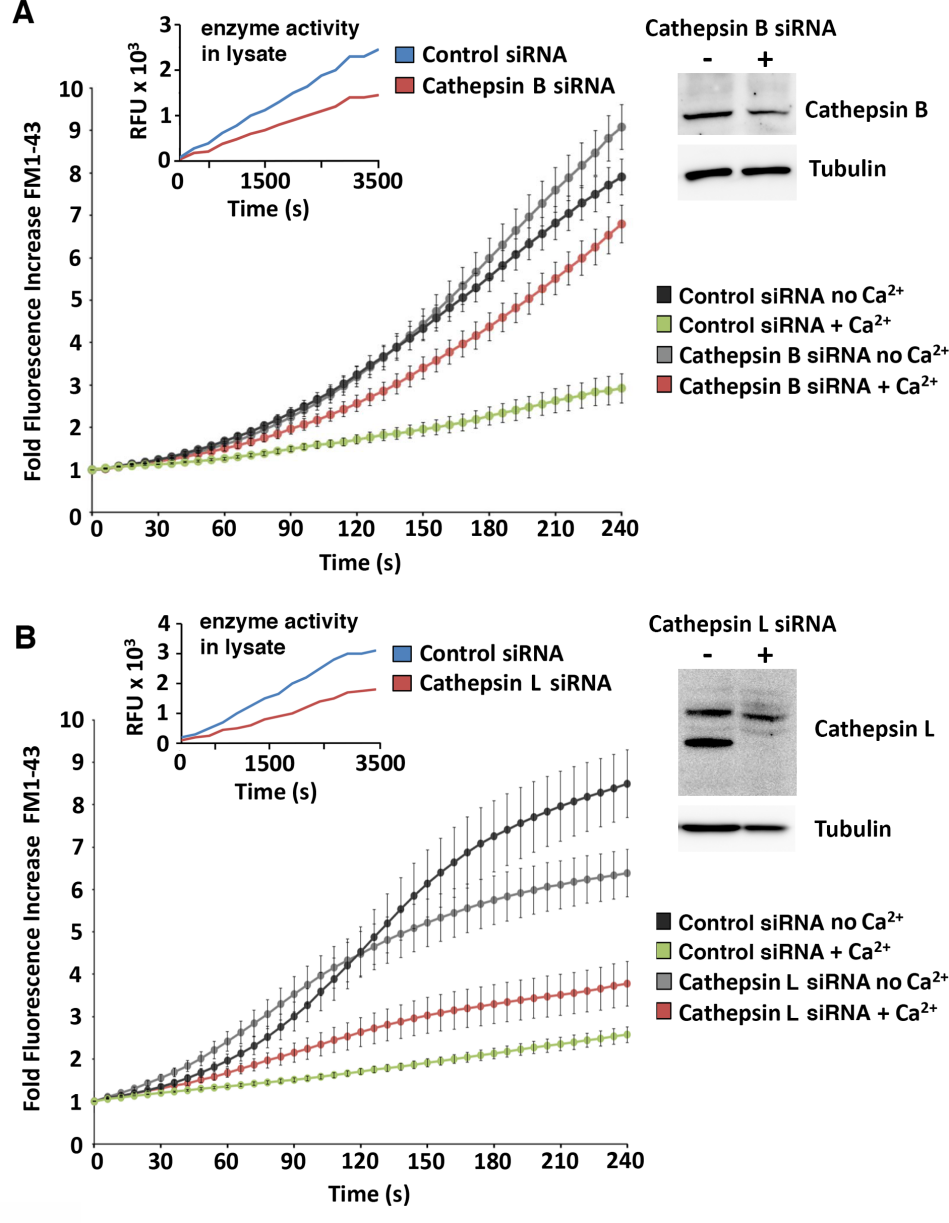
PM repair is inhibited in cells deficient in the lysosomal cysteine proteases cathepsin B or cathepsin L. Time-lapse live imaging of FM1-43 influx was performed in HeLa cells exposed to SLO in the presence or absence of Ca^2+^, 48 h after transfection with control or cathepsin B siRNA **(A)** or 72 h after cathepsin L **(B)** siRNA. The insets show kinetic enzyme activity determinations using a specific fluorogenic substrate (left) or the protein levels of each enzyme detected by immunoblot with specific antibodies (right). Anti-tubulin antibodies were used in the same samples as loading controls. The data are representative of two independent experiments.

**Fig 7:**
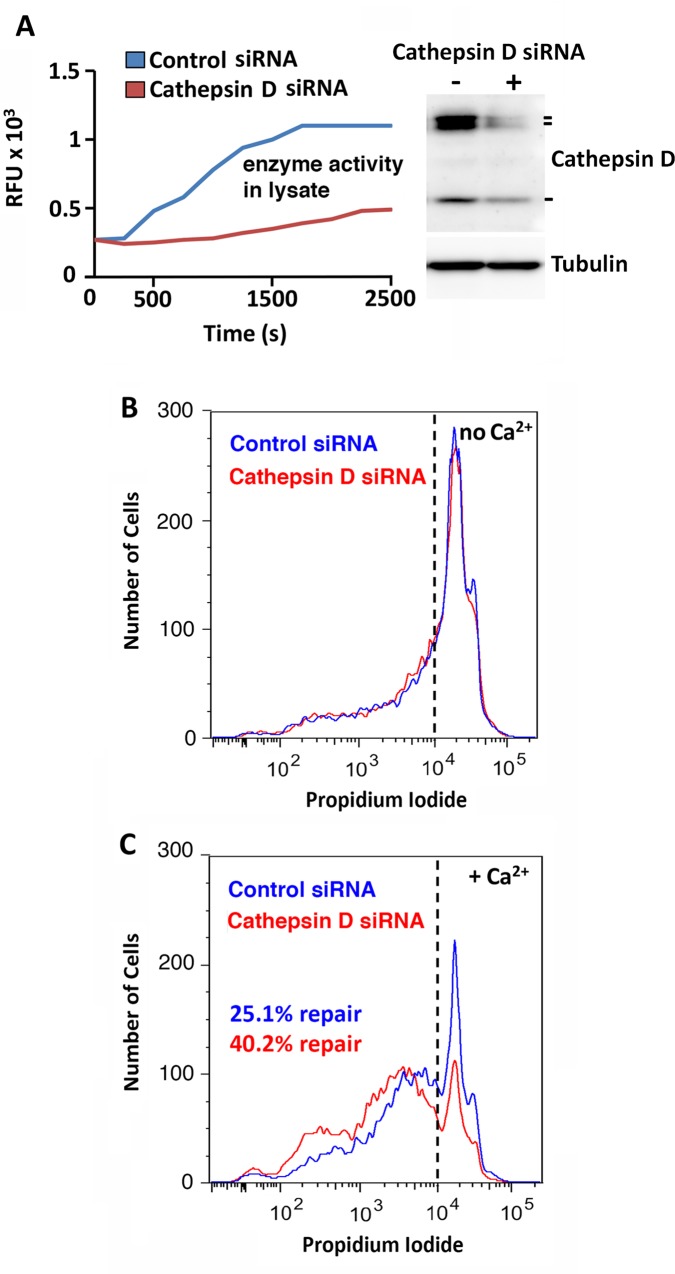
Deficiency in the lysosomal aspartyl protease cathepsin D facilitates PM repair. **(A)** Cathepsin D activity determined kinetically using a specific fluorogenic substrate (left) and cathepsin D protein (precursor and mature forms) levels detected by immunoblot with specific antibodies (right) in HeLa cell lysates 36 h after transfection with control (blue) or cathepsin D (red) siRNA. Anti-tubulin antibodies were used in the same samples as loading controls. **(B,C)** FACS quantification of PI staining in HeLa cells treated with control (blue) or cathepsin D (red) siRNA after wounding by scraping in the absence **(B)** or presence **(C)** of Ca^2+^. After a resealing period of 2 min at 37°C the cell population was stained with PI and analyzed by FACS. The dashed line indicates the gating based on the Ca^2+^-free permeabilization control. The data are representative of two independent experiments.

To directly address the role of major lysosomal proteases in the regulation of PM repair, we used RNAi to deplete HeLa cells in cathepsins B, L and D and performed wounding and resealing assays. To make sure that our siRNA procedure reduced the amount of active enzyme in the cells, in addition to western blots we also performed specific enzyme activity assays in the lysates. Closely mirroring what we previously observed with E64 and pepstatin-A (Figs 3 and 4), siRNA-mediated depletion of cathepsins B and L activity reduced PM repair (Fig 6A and 6B), whereas cathepsin D activity depletion improved the ability of cells to reseal (Fig 7A–7C). Thus, although we cannot rule out that cytosolic proteases released from injured cells also contribute to the observed extracellular proteolytic activity and influence PM repair [21, 42], our results implicate the lysosomal cysteine proteases cathepsin B and L in facilitating PM resealing, and cathepsin D as a negative regulator of the process.

### ASM Is Proteolytically Activated Extracellularly and Binds to the Cell Surface, Where It Is Subsequently Downregulated by Cathepsin D

**Fig 8:**
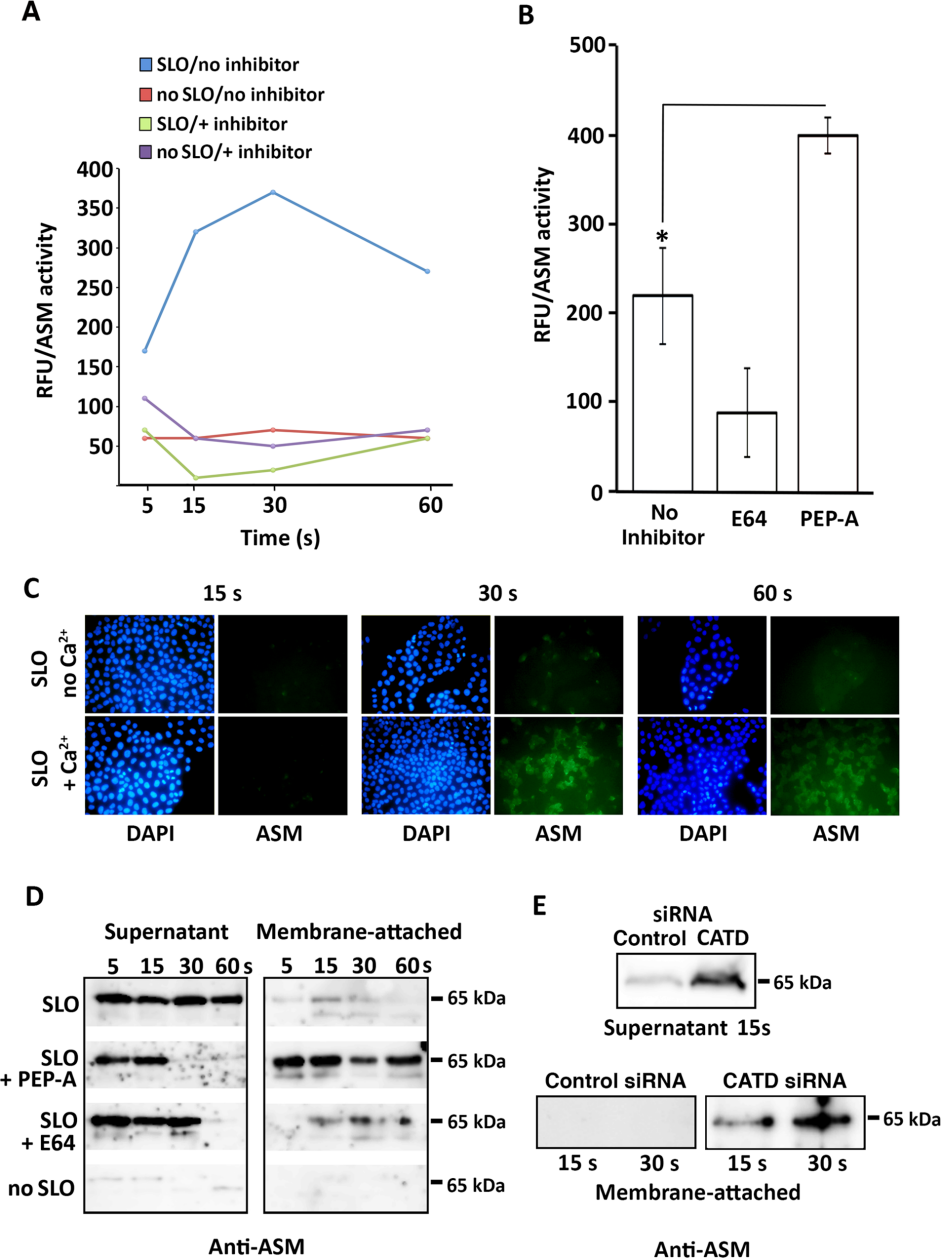
ASM secreted during cell injury associates with the PM and is proteolytically modulated. **(A)** Effect of protease inhibitors on ASM activity released from wounded cells. NRK cells were permeabilized with SLO (100 ng/ml) in Ca^2+^ media containing (green) or not (blue) a protease inhibitor cocktail and incubated at 37°C for the indicated time. As controls, cells not treated with SLO (NT) were incubated with (purple) or without (red) inhibitors. Supernatant samples were placed on ice, protease inhibitors were added and ASM activity was assayed. Similar results were obtained with HeLa cells (not shown). **(B)** Effect of inhibitors on ASM activity released from wounded cells. NRK cells were permeabilized with SLO (100 ng/ml) in Ca^2+^-containing media with 100 μM E64, 100 μM pepstatin-A (PEP-A) or no inhibitors, and incubated at 37°C for 30 s. The data represent the mean +/- SD of triplicate assays. * P = 0.039, Student’s t test. **(C)** Detection of cell-associated ASM during the first seconds after SLO wounding. NRK cells were permeabilized with SLO (100 ng/ml), incubated at 37°C in Ca^2+^-containing media for the indicated time, washed and immunofluorescence was performed with rabbit anti-ASM antibodies, followed by imaging under identical settings. **(D)** Detection of the active 65 kDa form of ASM in supernatant and membrane-associated fractions of wounded cells. NRK cells were permeabilized or not with SLO (100 ng/ml) with or without 100 μM E64 or 100 μM pepstatin-A for the indicated periods of time, and samples of the supernatant or of material removed from the cell surface by an acid wash were analyzed by Western blot with rabbit anti-ASM antibodies. The data are representative of at least three independent experiments. **(E)** Detection of the 65 kDa ASM form in the supernatant and membrane-associated fractions of wounded cells depleted or not in cathepsin D (CATD). NRK cells were permeabilized or not with SLO and analyzed as described in (D). The data are representative of at least three independent experiments.

The lysosomal lipase ASM plays an important role in PM repair, and can rescue membrane resealing defects when added extracellularly in the absence of Ca^2+^ [33]. Our present results, showing that abundant lysosomal proteases are rapidly secreted from wounded cells and regulate PM repair, suggested that extracellular proteolysis might modulate ASM activity. To investigate this possibility, we performed SLO wounding and repair assays in the presence or absence of a cocktail of protease inhibitors, and followed the activity of extracellular ASM during the process using a specific assay (specificity controls for the ASM activity assay are shown in S2 Fig). In the absence of inhibitors the activity of ASM detected in the supernatant increased sharply during the first 30 seconds after SLO wounding, followed by a decrease (Fig 8A, blue curve). On the other hand, in the presence of a cocktail of protease inhibitors the extracellular ASM activity was strongly reduced (Fig 8A, green curve). Very low levels of ASM activity were also detected in the supernatant of non-wounded cells, in the presence or absence of inhibitors (Fig 8A, red and purple curves). As an additional control, after collecting the cell supernatant all samples were immediately placed on ice with the protease inhibitor cocktail. Thus, proteolytic events occurring extracellularly during the first few seconds after PM wounding appear to be responsible for a substantial increase in active ASM. When the protease inhibitor cocktail was replaced by E64, a small but very reproducible decrease on the amount of active ASM released was also observed (Fig 8B), suggesting that cysteine proteases participate in extracellular ASM activation but may not be the only proteases involved in this step [43]. Strikingly, when the same assay was performed in the presence of the aspartyl protease inhibitor pepstatin-A, the ASM activity detected in the supernatant of wounded cells was further increased, when compared to cells not exposed to inhibitors (Fig 8B). This result suggests that one or more aspartyl proteases released extracellularly during the first 30 s after PM wounding participate in inactivating ASM activity. Since this effect was not seen with the protease inhibitor cocktail that also contains pepstatin-A, aspartyl proteases are likely to act downstream of the ASM activation step by enzymes with a different specificity.

To determine if ASM released from lysosomes of wounded cells becomes associated with the outer leaflet of the PM, where its substrate sphingomyelin is present, we performed immunofluorescence microscopy with anti-ASM antibodies. We found that after wounding with SLO in the presence of Ca^2+^, a condition that triggers exocytosis of lysosomes and ASM release [33], ASM gradually accumulates on the cell surface. Association of ASM with the cell surface was markedly reduced in the absence of extracellular Ca^2+^, consistent with the conclusion that ASM is delivered extracellularly through Ca^2+^-triggered exocytosis of lysosomes (Fig 8C). We proceeded to examine the supernatant and cell surface-associated fractions for presence of the 65 kDa form of ASM, which was previously shown to correspond to the active form of this enzyme [43]. In the absence of protease inhibitors, anti-ASM antibodies recognized a 65 kDa band mostly in the cell supernatant, with only small amounts detected in association with the cell surface in the first few seconds after wounding (Fig 8D, SLO). In contrast, when pepstatin-A was present there was a marked shift in the distribution of the active 65 kDa form of ASM. At very early time points the 65 kDa protein was detected in the supernatant, but subsequently there was a marked accumulation in the cell-associated fraction (Fig 8D, PEP-A). This observation is very consistent with the previously demonstrated role of ASM in promoting endocytosis and PM repair [33, 16], and with our findings of enhanced resealing in cells wounded in the presence of pepstatin-A (Figs 3 and 4). Under the same conditions, the cysteine protease inhibitor E64 had a small but reproducible effect of promoting accumulation of the 65 kDa form of ASM at the cell surface, particularly at later time points (Fig 8D, E64). Importantly, RNAi-mediated silencing of the aspartyl lysosomal protease cathepsin D had an effect very similar to what was observed with its inhibitor, pepstatin-A–accumulation of the 65 kDa ASM form in the cell-associated fraction (Fig 8E). Taken together with the enhanced ability of cells to reseal under conditions that interfere with cathepsin D activity, these results suggest that at least one of the mechanisms by which the lysosomal aspartyl protease cathepsin D acts as a negative modulator of PM repair is by down-regulating the membrane-associated active form of ASM.

## Discussion

**Fig 9:**
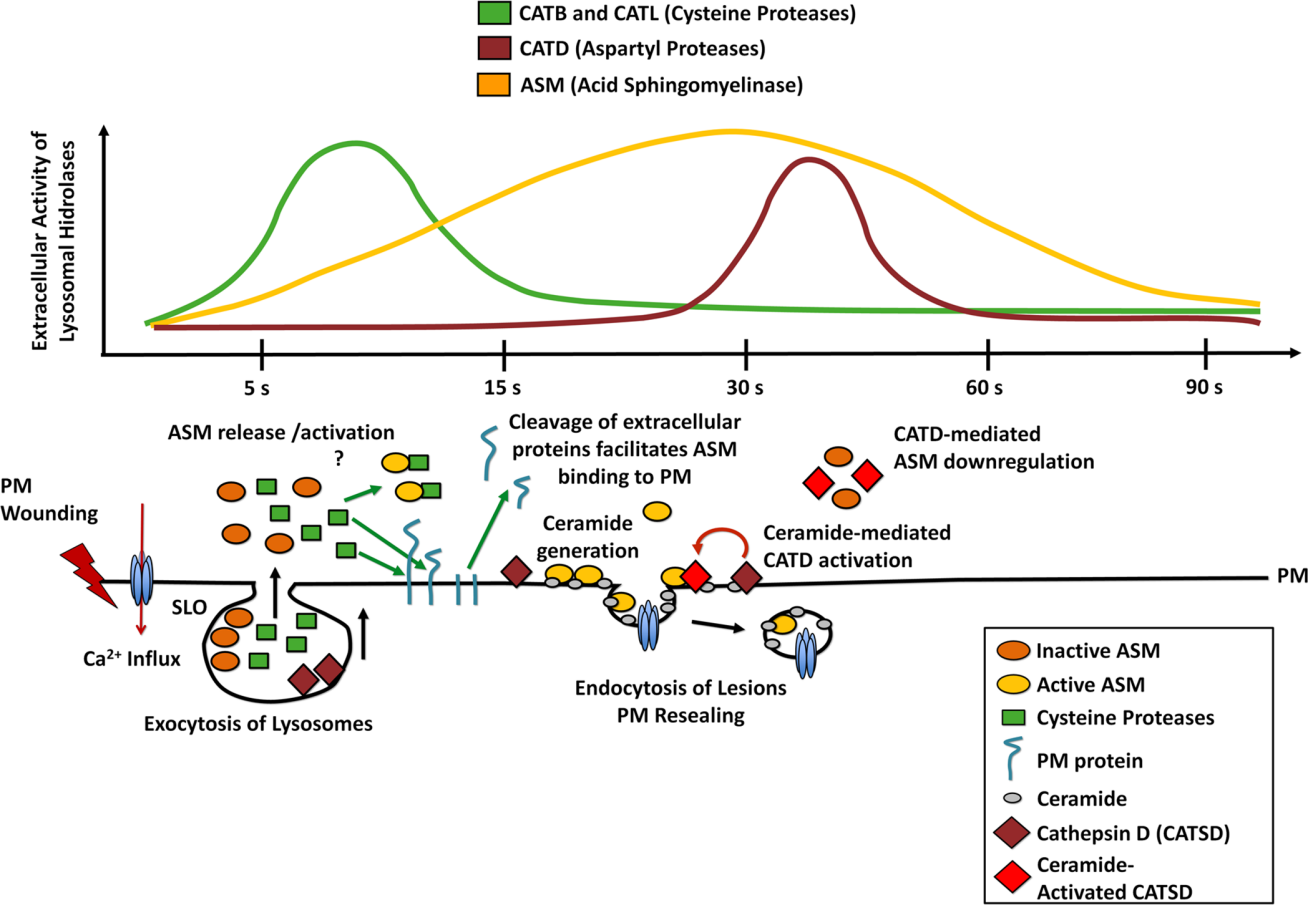
Proposed mechanism for rapid modulation of PM repair by secreted lysosomal proteases. Ca^2+^ influx through wounds in the PM rapidly triggers exocytosis of lysosomes, releasing the proteases cathepsins B, L and D along with the lipase ASM. Cathepsins B and L are active extracellularly 5–10 s after PM wounding, while cathepsin D activity appears after a delay of 30–60 s. At early time points, cathepsins B and L (and possibly additional cysteine proteases) may cleave cell surface proteins and contribute to membrane access and/or activation of ASM. ASM hydrolizes sphingomyelin on the outer leaflet of the PM generating ceramide, which promotes lesion endocytosis and PM repair [13, 30]. ASM-generated ceramide may enhance cathepsin D activity, which plays a role in down-regulating ASM. Around 1 min after wounding the PM integrity is restored, and lysosomal hydrolases are no longer active extracellularly.

Earlier investigations of the mechanism of PM repair have mostly focused on intracellular events, occurring at the cytoplasmic side of the membrane wound. In this study, we found that extracellular activity of proteases released from lysosomes play a role in regulating PM repair. First, we detected an increase in extracellular proteolysis during the first seconds after cell wounding and found that this process promotes PM repair. Second, active cysteine lysosomal proteases were released into the supernatant shortly after cell wounding, coinciding with an E64-sensitive solubilization of cell-associated proteins observed during the same period. Third, inhibition of cysteine proteases or RNAi-mediated silencing of the cathepsins B and L interfered with the ability of cells to reseal after injury, suggesting that these lysosomal cysteine proteases may facilitate PM repair by hydrolyzing proteins associated with the cell surface. Fourth, aspartyl protease inhibitors or RNAi-mediated silencing of cathepsin D enhanced PM repair, an effect that was associated with cell surface accumulation of active ASM, a lysosomal enzyme that promotes PM repair [20, 33]. Collectively, our results reveal that proteases released from wounded cells through lysosomal exocytosis can regulate PM repair, apparently by initially facilitating the process and subsequently down-regulating it (a proposed mechanism is shown in Fig 9).

The extracellular release of lysosomal proteases that we detected agrees with widespread earlier observations showing that elevations in cytosolic free Ca^2+^, resulting from signaling or PM injury events, trigger exocytosis of lysosomes [12, 27, 34, 44, 45]. However, to our knowledge this study is the first demonstration that exocytosis of lysosomal proteases occurs within a time scale of seconds after PM injury. We found that 5–7% of the total cellular content of the cysteine proteases cathepsin B and L are released into the medium, reaching maximum levels within just 5 seconds after wounding (Fig 5). Our results also support the notion that these secreted lysosomal proteases function extracellularly. When the cysteine protease inhibitor E64 was present in the medium during the short period of a few seconds between PM wounding and resealing, solubilization of cell surface proteins (Fig 2B) and PM repair (Figs 3B and 4B and 4F) were inhibited. PM repair was also impaired in cells specifically depleted in cathepsin B or L (Fig 6), further implicating abundant lysosomal cysteine proteases. Our results are in agreement with an earlier study in transected rat septal neurons, which concluded that membrane resealing was inhibited by inhibitors of cysteine proteases, and markedly enhanced by exposure to the exogenous proteases papain, trypsin and dispase [46].

Extensive evidence demonstrates that lysosomal cysteine proteases released into the extracellular milieu promote degradation and remodeling of extracellular matrix proteins [40, 41]. Such evidence includes direct visualization of extracellularly active cysteine cathepsins using fluorescently quenched activity-based probes [47–49]. We made similar observations in cells wounded with SLO. In the presence of extracellular Ca^2+^, a condition required for lysosomal exocytosis and PM repair, proteolytic de-quenching of DQ-BSA was observed in association with the cell surface, as early as 30 s after cell wounding (Fig 1B). Several mechanisms have been proposed to explain how acidic lysosomal hydrolases can function extracellularly. Cysteine cathepsins remain active for short periods in neutral pH [50, 51] and are relatively resistant to oxidative stress [52]. Furthermore, cathepsins are often secreted as inactive zymogens that can be activated extracellularly, a process that can extend their life outside the lysosomal environment [53]. Moreover, in a number of situations and particularly under pathological conditions, the pH of the local microenvironment is often acidified [40], a process that has been linked to translocation of the vacuolar V1H(+)-ATPase to the cell surface during lysosomal exocytosis [36].

Earlier studies from our laboratory showed that exocytosis of the lysosomal enzyme ASM plays an important role in PM repair [20]. ASM-deficient cells have a marked defect in PM repair when wounded, and purified human ASM or bacterial sphingomyelinase restore resealing when added extracellularly [33]. This finding is in agreement with earlier evidence showing that ASM can promote hydrolysis of sphingomyelin on the outer leaflet of PM [54, 55]. Based on our present results, we propose that degradation of PM-associated proteins by lysosomal cysteine cathepsins [40] promotes PM repair by facilitating the access of ASM to its substrate, sphingomyelin, on the outer leaflet of the PM [54, 56]. In support of this view, cell exposure for just a few seconds to the cysteine protease inhibitor E64 was sufficient to inhibit the solubilization of cell-associated proteins observed after cell wounding, and PM repair. The rapid kinetics of PM repair, which is completed within about 30 s after wounding [15, 57], may be relevant for the process by which secreted lysosomal proteases regulate PM repair. The numerous mechanisms that are in place to limit the extracellular activity of lysosomal proteases, such as dependence on acidic pH/reducing conditions and the abundance of endogenous inhibitors such as cystastins, kininogens, serpins and alpha-2-macroglobulin [58], may restrict the PM remodeling events involved in resealing to the very first few seconds after wounding, ensuring that continued surface protein degradation, sphingomyelin hydrolysis and endocytosis do not cause deleterious effects.

Our measurements of the kinetics of lysosomal enzyme secretion revealed that active cathepsin D is detected in the supernatant of wounded cells with a delay, when compared to cathepsins B and L (Fig 5). After synthesis in the rough ER and removal of the signal peptide, procathepsin D is targeted to lysosomes, where additional proteolytic processing events generate the mature active form of the enzyme [59][60]. Interestingly, ceramide, which is the product of sphingomyelin hydrolysis by ASM [61], can activate cathepsin D [62, 63]. We observed a gradual accumulation of active ASM extracellularly, with maximum activity reached between 15 and 30 seconds after cell wounding (Fig 8A). Thus, we suggest that the delayed detection of active cathepsin D in the extracellular medium after cell wounding may have resulted, at least in part, from activation of this aspartyl protease by ASM-generated ceramide (Fig 9). We cannot, however, rule out other potential explanations for the delayed extracellular detection of active/soluble cathepsin D, such as activation by other factors [40], the known association of cathepsin D to intracellular membranes [64, 65] and/or extracellular proteolytic processing of the pro-enzyme, which is co-secreted with active cysteine proteases [60]. The involvement of additional regulatory events in the generation of active soluble cathepsin D is in agreement with the smaller fraction of the total cellular activity of cathepsin D that we observed in the supernatant of wounded cells, when compared to the cysteine proteases cathepsins B and L.

Regardless of the explanation for the delayed release of active cathepsin D after cell wounding, it is noteworthy that the time point when maximum amounts of this aspartyl protease were detected in the medium (Fig 5) coincided with a decrease in extracellular ASM activity (Fig 8A). When the potent aspartyl protease inhibitor pepstatin-A was added during cell wounding, within a few seconds more ASM activity was detected extracellularly (Fig 8B), an observation that correlated with accumulation of the 65 kDa form of ASM (identified as the active lysosomal form of ASM [43]) on the cell surface (Fig 8D). A similar increase in membrane-associated 65 kDa ASM was observed after RNAi-mediated silencing of cathepsin D (Fig 8E). Conversely, when pepstatin-A was not present there was a strong reduction in cell-associated 65 kDa ASM (Fig 8D). Given the demonstrated affinity of cathepsin D for ceramide and other sphingolipids that are abundant on the outer leaflet of the PM [63, 65], it is conceivable that release and/or activation of cathepsin D from wounded cells participates in the down-regulation of ASM, blocking its activity on the cell surface (Fig 9). Given the major cell signaling role of ceramide [66], inactivation of the cell-associated ASM by cathepsin D following the completion of PM repair may play an important role in avoiding harmful consequences of excessive ceramide production.

Our results also suggest that proteases released from wounded cells may be involved in processing events that activate ASM extracellularly. Protease inhibitors added during cell wounding and repair block detection of ASM activity, but not when inhibitors are added to the culture medium after it was removed from cells (Fig 8A). One or more cysteine proteases may be, at least in part, responsible for such processing events, because E64 caused a small but consistent reduction in the ASM activity detected extracellularly (Fig 8B). Removal of a single thiol group of the C-terminal cysteine residue (Cys^629^), a change that would not have caused a significant shift in migration of the 65 kDa form detected in our assays, was reported to increase ASM activity [67]. It will be of interest to determine if lysosomal cysteine proteases, such as cathepsin B that has both endo and exopeptidase activity [68] play a role in this process.

Although much still remains to be understood regarding the involvement of lysosomal proteases released from wounded cells in the PM repair process, this study identified several specific steps where this process comes into play. These findings significantly expand our understanding of the complex molecular mechanism of PM repair, and clarify the physiological significance of previous reports of lysosomal proteases acting extracellularly. A better understanding of this process may facilitate the future development of strategies for treating serious human diseases, such as forms of muscular dystrophy linked to defective PM repair [69], and skin conditions associated with defects in extracellular matrix remodeling by lysosomal proteases [70].

## Supporting Information

S1 Fig Protease inhibitors rapidly modulate the repair of SLO wounds. NRK cells were wounded with SLO and time-lapse live imaging of FM1-43 influx was performed in the presence or absence of the following protease inhibitors: **(A)** E64, inhibitor of cysteine proteases (300 μM); **(B)** Pepstatin-A inhibitor of aspartyl proteases (100 μM); **(C)** AEBSF, inhibitor of serine-proteases (100 μM). Decrease in the influx of FM1-43 influx reflects PM repair. Bars: 10 μm.
(TIF)

S2 Fig Specificity of enzymatic assays. **(A)** Cathepsin B, L and D activity in lysates of HeLa cells previously treated with cathepsin B siRNA for 24 (blue) or 48 h (orange), determined using specific fluorogenic substrates for each enzyme. The only reduction observed was in cathepsin B activity. **(B)** Cathepsin B, L and D activity in lysates of HeLa cells previously treated with cathepsin L siRNA for 24 or 48 h, determined using specific fluorogenic substrates for each enzyme. The only reduction observed was in cathepsin L activity. **(C)** Cathepsin B, L and D activity in lysates of HeLa cells previously treated with cathepsin D siRNA for 24 or 48 h, determined using specific fluorogenic substrates for each enzyme. The only reduction observed was in cathepsin D activity. **(D)** ASM activity in lysates of HeLa cells previously treated with ASM siRNA for 72 h determined at pH 5.0 (optimum pH for lysosomal acid sphingomyelinase-ASM) or pH 7.4 (optimal pH for cytosolic neutral sphingomyelinase) using specific fluorogenic substrates for sphingomyelinase activity. The only reduction observed was at pH 5.0, the condition that allows detection of ASM activity. **(E)** ASM activity released through lysosomal exocytosis from NRK or HeLa cells treated with control siRNA of ASM siRNA, wounded with SLO (200 ng/ml) for 30 s. The enzymatic activity was determined under the two pH conditions as described in (D). Sphingomyelinase activity was only detected at pH 5.0, consistent with the cell wounding-induced exocytosis of lysosomal ASM (and not cytosolic neutral sphingomyelinase) from wounded cells.
(TIF)
