# Supplementary figures and images for: Plasma Membrane Repair Is Regulated Extracellularly by Proteases Released from Lysosomes
Source: PLoS One. 2016 Mar 30;11(3):e0152583. doi: 10.1371/journal.pone.0152583 (PMC4814109; doi:10.1371/journal.pone.0152583)

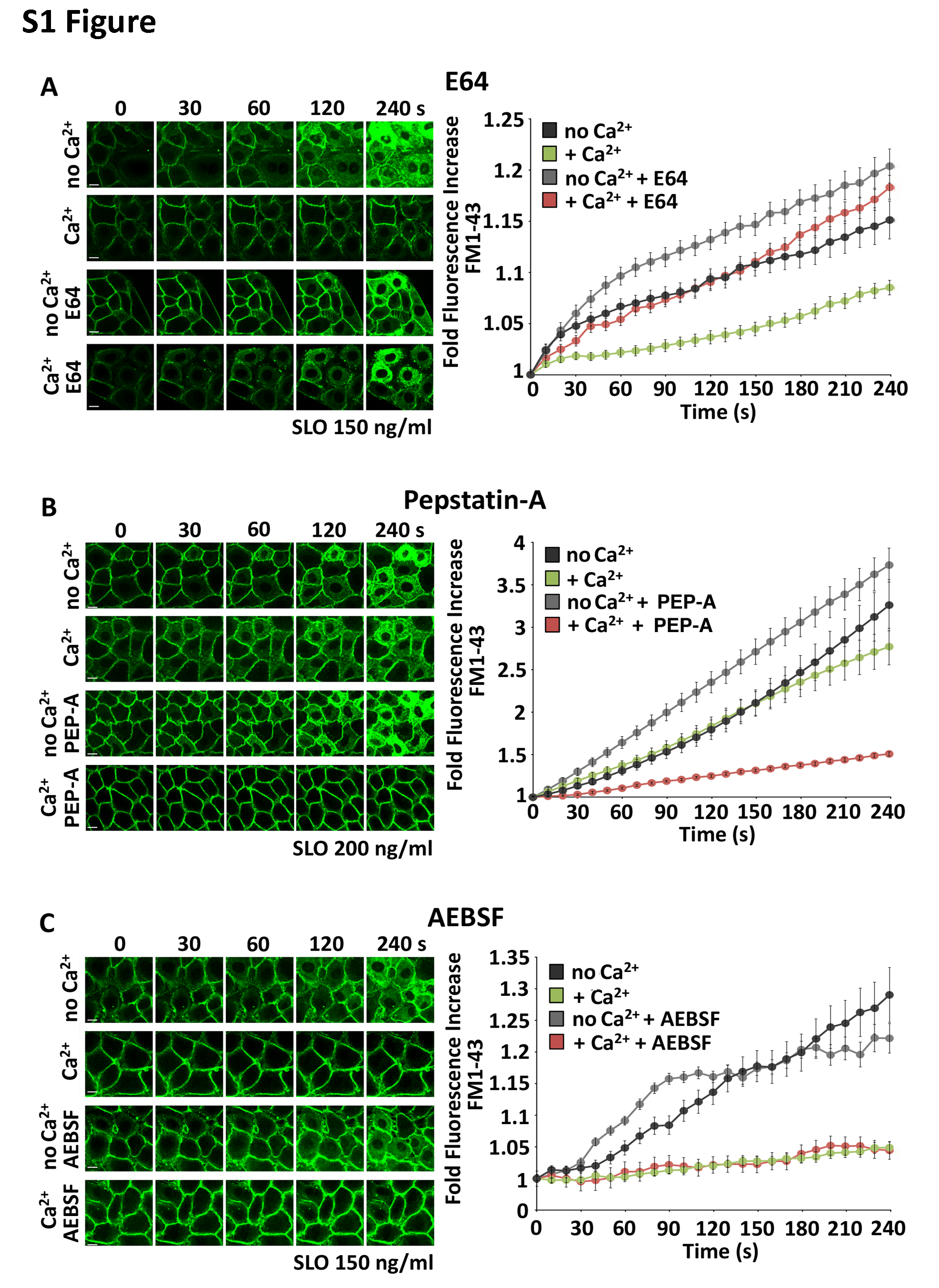

Supplement: S1 Fig — NRK cells were wounded with SLO and time-lapse live imaging of FM1-43 influx was performed in the presence or absence of the following protease inhibitors: (A) E64, inhibitor of cysteine proteases (300 μM); (B) Pepstatin-A inhibitor of aspartyl proteases (100 μM); (C) AEBSF, inhibitor of serine-proteases (100 μM). Decrease in the influx of FM1-43 influx reflects PM repair. Bars: 10 μm. (TIF) [file pone.0152583.s001.tif]

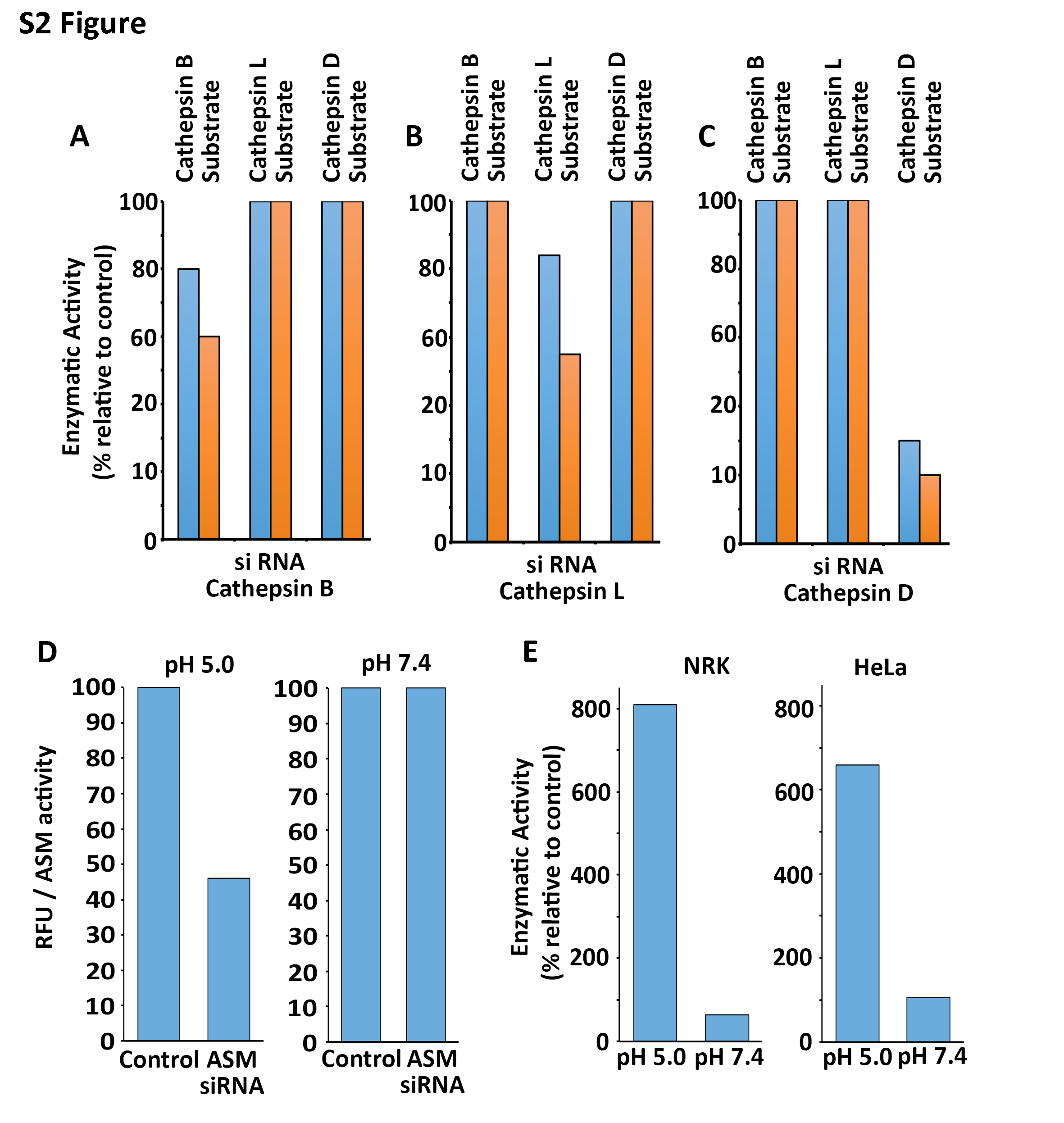

Supplement: S2 Fig — (A) Cathepsin B, L and D activity in lysates of HeLa cells previously treated with cathepsin B siRNA for 24 (blue) or 48 h (orange), determined using specific fluorogenic substrates for each enzyme. The only reduction observed was in cathepsin B activity. (B) Cathepsin B, L and D activity in lysates of HeLa cells previously treated with cathepsin L siRNA for 24 or 48 h, determined using specific fluorogenic substrates for each enzyme. The only reduction observed was in cathepsin L activity. (C) Cathepsin B, L and D activity in lysates of HeLa cells previously treated with cathepsin D siRNA for 24 or 48 h, determined using specific fluorogenic substrates for each enzyme. The only reduction observed was in cathepsin D activity. (D) ASM activity in lysates of HeLa cells previously treated with ASM siRNA for 72 h determined at pH 5.0 (optimum pH for lysosomal acid sphingomyelinase-ASM) or pH 7.4 (optimal pH for cytosolic neutral sphingomyelinase) using specific fluorogenic substrates for sphingomyelinase activity. The only reduction observed was at pH 5.0, the condition that allows detection of ASM activity. (E) ASM activity released through lysosomal exocytosis from NRK or HeLa cells treated with control siRNA of ASM siRNA, wounded with SLO (200 ng/ml) for 30 s. The enzymatic activity was determined under the two pH conditions as described in (D). Sphingomyelinase activity was only detected at pH 5.0, consistent with the cell wounding-induced exocytosis of lysosomal ASM (and not cytosolic neutral sphingomyelinase) from wounded cells. (TIF) [file pone.0152583.s002.tif]
